# The Touching Difference: Evidence for Stimulus-Response Binding Effects in Tactile Detection and Localization Performance but Not in Their Visual Counterparts

**DOI:** 10.5334/joc.476

**Published:** 2026-01-07

**Authors:** Lars-Michael Schöpper, Paula Soballa, Simon Merz, Christian Frings

**Affiliations:** 1 Department of Cognitive Psychology, University of Trier, 54286 Trier, DE

**Keywords:** Action control, Attentional orienting, Stimulus-response binding, modality dependence

## Abstract

According to action control theories, responding to stimuli leads to a binding of stimulus and response features into a common representation referred to as an event file. If any component of this event file repeats, information is retrieved and affects performance: While full repetition is beneficial, partial repetition leads to cost. These stimulus-response (S-R) binding effects have been found in very many experimental designs; yet, these effects are typically completely absent in visual detection and localization tasks. Recently, however, it has been found that contrary to vision, auditory detection and localization leads to binding effects, thus suggesting modality dependence. In the current study we aimed to extend this debate by comparing the visual with the tactile domain. Participants detected (Experiment 1) or localized (Experiment 2) visual targets of different color and tactile targets of different intensity and rhythm. In both detection and localization, we observed evidence of binding and retrieval in the tactile domain which was completely absent in the visual domain. The results highlight the previously suggested modality dependence for binding approaches in action control.

It is a hot evening in late summer and you enjoy a cold drink at the lake. Suddenly you notice a tickling feeling on your arm; you quickly realize a mosquito is about to bite you! You lash out trying to chase it away. From an action control perspective this bodily movement is considered an action as it is intentional and/or is executed with a specific goal in mind (Frings et al., 2020; 2024; Prinz, 1998). These simple actions are the subject of investigation by action control theories. According to the theory of event coding (TEC; Hommel et al., 2001) and the binding and retrieval in action control (BRAC; Frings et al., 2020, 2024; see also Beste et al., 2023) framework, when executing such an action the movement and information of the to-be-acted upon stimulus are coupled into a short episodic memory trace, an event file (Hommel, 2004). Even information completely irrelevant for responding can be bound (Frings et al., 2007; Rothermund et al., 2005). Repeating any component of this event file retrieves the previous information, affecting performance. In case of complete repetition, this does not lead to any interference (e.g., Hommel, 1998) and responding can even become beneficial (potentially fueled by overall response repetition benefits; Frings et al., 2007). However, in case of partial repetition, costs emerge as the previous event file is retrieved but no longer valid. You thus should be faster in “responding” to the mosquito if it reappears on the same spot on your arm – however, if now a harmless ladybug lands there, your previous but now incorrect movement to the mosquito should be retrieved by the repeated tactile stimulation.

The resulting so-called stimulus-response (S-R) binding effect – the effect emerging from the processes of binding and retrieval that summarizes the partial repetition costs of response repetitions and changes – can be measured in prime-probe sequences in which response-relevant and -irrelevant features are orthogonally varied to repeat or change (Frings et al., 2007). For example, participants indicate the color of stimuli repeating or changing their location (e.g., Schöpper et al., 2020). If the location repeats this is thought to retrieve the previous response – beneficial if the same response is required but causing interference if another response is demanded. Yet, if the location changes, a response change is beneficial because this is not interfered by retrieval – in contrast to a response repetition in this case. However, binding effects have been observed for a number of response-irrelevant stimulus dimensions like color, letters, or orientation (e.g., Frings et al., 2007; Hommel, 1998). The underlying processes of binding and retrieval are thought to affect responding in many sequential designs such as conflict tasks (Davelaar & Stevens, 2009), priming (Henson et al., 2014), task switching (Koch et al., 2018), and more (see Frings et al., 2020).

Yet, when indicating the detection or location of sequentially presented visual stimuli, the pattern looks quite different. In these tasks stimuli can repeat or change their non-spatial identity like color or shape while participants are instructed to signal the detection (Kwak & Egeth, 1992; Schöpper & Frings, 2023, 2024; Schöpper et al., 2020) or localize (Hilchey et al., 2018; Schöpper & Frings, 2022, 2024; Schöpper et al., 2022a; Taylor & Donelly, 2002) stimuli repeating or changing their location. The setup can be identical to prime-probe sequences as discrimination tasks with orthogonally varied locations (e.g., Schöpper et al., 2020; Schöpper & Frings, 2022), however, the response attribute is different: For example, in a localization task participants indicate if a target that repeats or changes its location as well as its non-spatial identity (e.g., color) is to the left or right of a fixation cross. In a detection task, the same key is pressed for every stimulus irrespective of location or non-spatial identity. Here, effects of binding and retrieval are typically completely absent (Schöpper & Frings, 2024; for an overview of attentional orienting designs, see Huffman et al., 2018) and only inhibition of return (IOR; Posner et al., 1985; see Klein, 2004) is observed. In other words, costs emerge for target location repetitions from prime to probe, but non-spatial feature repetitions or changes typically do not have any impact, nor do they interact with location repetitions/changes.

IOR has initially been explained by attention being captured by a stimulus followed by the inhibition of said location (Posner & Cohen, 1984; Posner et al., 1985). However, since its initial observations (see also Berlucchi et al., 1981; Maylor & Hockey, 1985), IOR has been argued to be the result of perceptual and motor processes (e.g., Taylor & Klein, 2000), the latter often discussed in the context of involving oculomotor activation (for a review, see Klein & Hilchey, 2011). Others have argued that IOR results from a stimulus being bound into an object file (Kahneman et al., 1992) and when this stimulus repeats, it is perceived as its former activation – detection costs occur (Lupiáñez, 2010; Lupiáñez et al., 2013; Martín-Arévalo et al., 2013). Irrespective of its exact origins or underlying mechanisms (see also Berlucchi, 2006; Dukewich & Klein, 2015), even the baseline IOR effect – a location repetition cost – is in contrast to binding and retrieval approaches, as these do not expect interference by repetition of information (for a discussion, see Schöpper & Frings, 2022). Yet, it is possible that IOR and S-R binding effects co-occur (e.g., Hilchey et al., 2018; Hommel, 1998, 2004; Schöpper & Frings, 2025; Schöpper et al., 2020, 2022b).

Several theories have been developed why binding effects in visual detection and localization procedures are absent (for discussions, see also Schöpper et al., 2020; Schöpper & Frings, 2024). Huffman et al. (2018) argued that it is the lack of attention towards non-spatial identity (Huffman et al., 2018); in line with that, attention to certain features increases retrieval thereof (e.g., Memelink & Hommel, 2013; Moeller & Frings, 2014) and effects can be absent if this attentional allocation is absent (Singh et al., 2018; 2025). Schöpper et al. (2022a; see also Geissler et al., 2024) argued that in detection and localization procedures responses are based on initial identification of targets, that is, based on direct visuomotor mechanisms (e.g., Fournier et al., 2015; Wiediger & Fournier, 2008) and spatial compatibility between stimulus and response (Kornblum et al., 1990). Simply stated, there is no need to translate a non-spatial feature (e.g., the color red) into a spatially-defined response, because the spatial feature already allows to give a response: The response can be executed directly with spatial correspondence (in case of localization task: a left or right target demands a left or right response; in case of a detection task: any target demands the same response). Crucially, binding and retrieval typically only occurs when this translatory stage takes place (e.g., Geissler et al., 2024; Schöpper & Frings, 2022, 2024; Schöpper et al., 2022a).

Yet, there are some effects of repeating (non-)spatial information in detection and localization procedures, although these are scarce. For finding effects congruent with binding approaches, specific response mappings are required, for example, pre-cued spatial responses (Chao & Hsiao, 2021; Chao et al., 2022), if space has to be processed prior responding (i.e., give detection response only if target is at a certain location; Hilchey et al., 2020), or if the location has to be “translated” into a spatially-opposing response (e.g., upper left response for lower right target; Geissler et al., 2024; Schöpper et al., 2022a). Alternatively, variations on feature dimension levels lead to effects of binding and retrieval (e.g., changes not from red to blue targets, but from color to orientation targets; Schöpper et al., 2024) suggesting that not individual features, but rather feature dimensions are bound to responses (see “dimension weighting account”, Found & Müller, 1996; Müller et al., 1995). Findings incongruent with binding approaches (for discussions see Schöpper & Frings, 2022, 2024; Schöpper et al., 2022a), are reflected in the observation of non-spatial IOR (Law et al., 1995) at location repetitions (Chao et al., 2020; Hu et al., 2011, 2013; Schöpper et al., 2022a); these costs for repeating all information at the same location (in a sense “full repetition costs”) have been attributed to a detection cost for repeated information (Lupiáñez, 2010; Lupiáñez et al., 2013; Martín-Arévalo et al., 2013).

Against this background of mostly absent binding effects in visual detection and localization performance, a striking modality difference has been identified: When participants are asked to signal the detection (Mondor & Leboe, 2008; Schöpper & Frings, 2023) or location (Dyson, 2010; Schöpper et al., subm.) of sequentially presented *auditory* stimuli with repeating or changing task-irrelevant pitch, effects of binding and retrieval are observed. This modality difference has been attributed to worse spatial resolution in the auditory compared to visual modality (e.g., Loomis et al., 1998) which might increase task difficulty which in turn boosts retrieval (Geissler et al., 2024), or to the auditory stimulus itself being alerting (van der Lubbe & Postma, 2005) and hard to ignore (Spence, Ranson, & Driver, 2000).

To summarize, effects of binding and retrieval are mostly absent in visual detection and localization tasks, but present in their auditory counterparts. Why this difference specifically emerges is not fully understood – does it hinge on visual processing being special in terms of binding and retrieval? Or is it due to the auditory domain imposing specific constraints on processing in terms of binding and retrieval? It thus seems important to investigate a third modality to fully understand the underlying mechanisms. The tactile domain is predestined to serve this function.

The question then emerges how tactile stimuli are processed in terms of sequential responding. On the one hand, responding to tactile stimuli shares similarities with that for auditory stimuli for which binding effects can be observed irrespective of task type (e.g., Schöpper & Frings, 2023): Mondor and Leboe (2008) observed IOR in an auditory cue-target design (i.e., only signal the detection of the second stimulus), whereas an auditory target-target design (i.e., signal the detection of both stimuli) showed no IOR but a frequency repetition benefit. The authors attributed this to retrieval in case of full repetition (for discussions, see also Schöpper & Frings, 2023; Schöpper et al., subm.). Such differences in strength of IOR when comparing cue-target and target-target sequences can also be seen with tactile stimuli. When comparing both trial types, Poliakoff et al. (2002) observed that IOR was reduced for the target-target task. Thus, responding in a tactile target-target sequence might be affected by retrieval due to response repetition which in turn reduces IOR (Hilchey, et al., 2018; Schöpper & Frings, 2025). In line with that, attention towards tactile stimuli has been found to be linked to motor cortex activity, suggesting a direct link for action preparation (Galazky et al., 2009). This might suggest that tactile stimuli are processed different than visual stimuli in terms of binding and retrieval – and share more similarities with processing in other domains such as audition.

On the other hand, IOR for tactile stimuli has been observed (Jones & Forster, 2014; Lloyd et al., 1999; Spence, Lloyd, et al., 2000), even if the modality switches from the sequentially presented stimuli (Spence, Lloyd, et al., 2000). IOR for non-spatial identity has been discussed in the context of tactile stimuli as well (Cohen et al., 2005). That response repetition benefits reduce IOR in target-target compared to cue-target designs has also been observed and discussed in the visual domain (Welsh & Pratt, 2006). Regarding discrimination tasks, tactile stimuli have been found to lead to S-R binding effects (Moeller & Frings, 2011; Zmigrod et al., 2009). This might suggest that tactile stimuli are processed just as those of the visual domain with regard to binding and retrieval.

## Current study

The present study was designed to investigate whether detecting and localizing tactile stimuli leads to effects of binding and retrieval. For that matter, we asked participants to signal the detection of tactile stimuli on their left arm with a key press (Experiment 1) or the location of the same stimuli with two different key presses (Experiment 2). In both experiments, tactile stimuli could repeat or change their location as well as repeat or change their rhythm and intensity. This was compared with a visual detection (Experiment 1) and localization (Experiment 2) task with visual targets repeating or changing their location on a screen while orthogonally varying their color.

**Figure 1:**
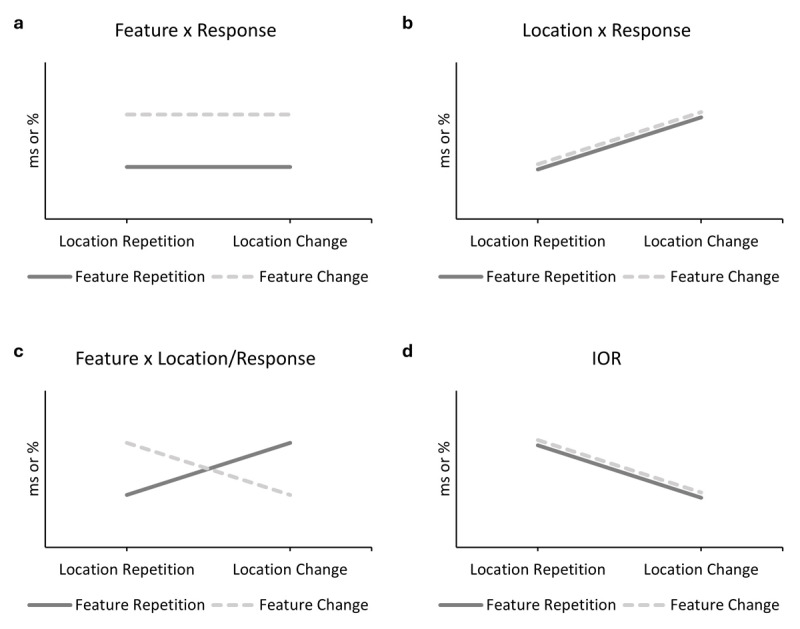
Hypothetical effects of feature relation and location relation. *Note*. In a detection task in which the response always repeats, a binding effect can be derived from a benefit (in reaction times in ms and/or in error rates in %) of **a)** (non-spatial) feature repetition or **b)** location repetition: Repeating the feature/location retrieves the previous response. Such a non-spatial feature repetition benefit has been found in auditory detection (Mondor & Leboe, 2008; Schöpper & Frings, 2023). However, an **c)** interaction of non-spatial feature relation and location relation marked by partial repetition costs irrespective of repeating the response is also theoretically possible. In a localization task, a binding pattern is derived from the interaction of non-spatial feature relation and location relation as well due to the location being confounded with the response. Such a pattern has been observed in auditory localization (Dyson, 2010; Schöpper et al., subm.) but in the visual domain only under quite specific setups (Schöpper et al., 2022a, 2024). Crucially, in visual detection (e.g., Huffman et al., 2018; Kwak & Egeth, 1992; Schöpper & Frings, 2023, 2024; Schöpper et al., 2020) and localization (e.g., Hilchey et al., 2018; Huffman et al., 2018; Schöpper & Frings, 2022, 2024; Schöpper et al., 2022a, 2024; Taylor & Donelly, 2002) performance these binding patterns are typically completely absent and d) IOR is observed – a location repetition cost.

If tactile stimuli in detection and localization are processed in terms of binding and retrieval just as visual stimuli, we would expect no effects of binding and retrieval, but only IOR (see Lloyd et al., 1999; Spence, Lloyd, et al., 2000), that is a cost for location repetition (see Figure 1d). If, however, tactile stimuli in this context are processed as auditory stimuli, we should find a pattern of binding and retrieval. If so, this would suggest that the visual modality is special regarding its role in binding and retrieval.

In Experiment 1, a binding pattern should be reflected in a main effect of non-spatial feature repetition or location repetition (see Figure 1a and b): The respective feature – rhythm or location – is bound to the response and upon repetition retrieved (as all responses in this design are response repetitions; see also Huffman et al., 2018; Schöpper et al., 2020; Schöpper & Frings, 2023, 2024). Alternatively, rhythm and location could be bound in a feature-feature binding irrespective of the response (see Figure 1c; e.g., Hommel, 1998; Kahneman et al., 1992; Treisman & Gelade, 1980). In Experiment 2 a binding pattern would be reflected in the interaction of non-spatial feature and response (see Figure 1c): The non-spatial feature should retrieve the response which is beneficial for full repetition, but causes interference if another response is demanded. In contrast, feature changes are only beneficial if the response also changes as no interference by partial repetition emerges.

Note that in Experiment 2 response and location are fully confounded: Repeating the response resembles repeating the location and changing the response resembles changing the location (see Schöpper & Frings, 2022; Schöpper et al., 2023). Thus, it is not possible to deduce if potentially emerging binding effects are due to response or location binding. However, we were interested in if a binding effect emerges at all, irrespective of being caused by response or location; neither of the latter leads to a binding pattern in the visual domain.

## Experiment 1: Detection task

### Methods

#### Participants

Binding effects are typically completely absent in visual detection and localization tasks (Huffman et al., 2018; Schöpper & Frings, 2023, 2024). However, in auditory detection performance the effect size can be medium (e.g., *d* = 0.37 in Schöpper & Frings, 2023; *d* = 0.51 in Mondor & Leboe, 2008, Experiment 1) and in auditory localization performance very strong (e.g., *d* = 1.44 averaged across pitch discrimination/pitch localization of Experiment 1, Dyson, 2010; *d* = 1.47 in Schöpper et al., subm.). We collected data of N = 30 participants, giving us a power of 1–ß = .85 for observing an effect size of at least *d* = 0.5 (α = .05, one-tailed; G*Power, Version 3.1.9.4; Faul et al., 2007). Thirty students from Trier University participated for course credit or a monetary reward of 10€ and gave written informed consent. One participant was excluded due to being a heavy outlier in overall error rate (12.76%) and a second participant was excluded due to being a heavy outlier in number of excluded trials (44.73%) and overall error rate (18.55%), leading to a final sample of 28 participants (21 females, 7 males, *M*_age_ = 25.29, *SD*_age_ = 2.99; age range: 20–32). All reported normal or corrected-to-normal vision.

#### Apparatus and Materials

The experiment was programmed in PsychoPy (Peirce et al., 2019). Participants were seated approximately 65 cm viewing distance from the screen. A white (R/G/B: 255/255/255) fixation cross (0.85 × 0.85 cm/ 0.75 × 0.75° of visual angle) was presented vertically centered and approximately 5.4 cm (4.75° of visual angle) to the right from screen center in both modality conditions. In the visual task, targets were red (R/G/B: 224/32/64) and blue (R/G/B: 64/64/192) dots, each 1.4 cm (1.23° of visual angle) in diameter, appeared on the left screen half approximately 5.4 cm (4.75° of visual angle) to the left from screen center in the upper or lower region. Vertical distance (center to center) between targets was 8.1 cm (7.13° of visual angle) and a target position on x-axis and fixation cross were 10.8 cm (9.5° of visual angle) apart (center to center), with an approximate diagonal distance of 11.53 cm (10.14° of visual angle) between an upper/lower target position and fixation cross (center to center). In the tactile task, tactors (model C-2, Engineering Accoustic, Inc.: 3 cm in diameter with a centrally located 0.76 cm diameter skin contactor) were placed 6 cm from the wrist and 6 cm from the elbow bend on the inner left arm of each individual participant. Then the arm was positioned with the tactors facing upwards on two soft arm rests, one positioned at the elbow, the other at the back of the hand. The arm rests were put so far apart that the participants could rest their arm comfortably but without touching the areas that were stimulated by the two tactors. The arm was positioned roughly in 90° angle from the body, with the hand facing the computer. Then, the arm was covered by a black box so that participants could not see the tactors. Further, participants wore earplugs (approximate noise reduction: 29 dB) and had to wear additional over-ear headphones on which brown noise was played (simultaneously presented frequency distribution with higher intensities at lower frequencies). This was done to eliminate any sounds to be perceived from the tactors that could influence performance. Tactile targets had a frequency of ~250 Hz and could be a constant vibration with high intensity or a rhythmic alteration of 40 ms vibration and 40 ms silence with low intensity, both with a duration of 200 ms. Thus, they were clearly distinguishable by intensity and rhythm.

#### Design

The experiment used a 2 (modality: visual vs. tactile) × 2 (location relation: repetition vs. change) × 2 (feature relation: repetition vs. change) within-subject design. In a detection task with continuous responding, the binding effect is derived from the main effect of location or feature, or from the interaction of location and feature. These can be further modulated by modality.

#### Procedure

Participants completed both the visual and tactile task. Task order was balanced and alternated with every participant. Each task started with the instruction being presented on screen.

**Figure 2:**
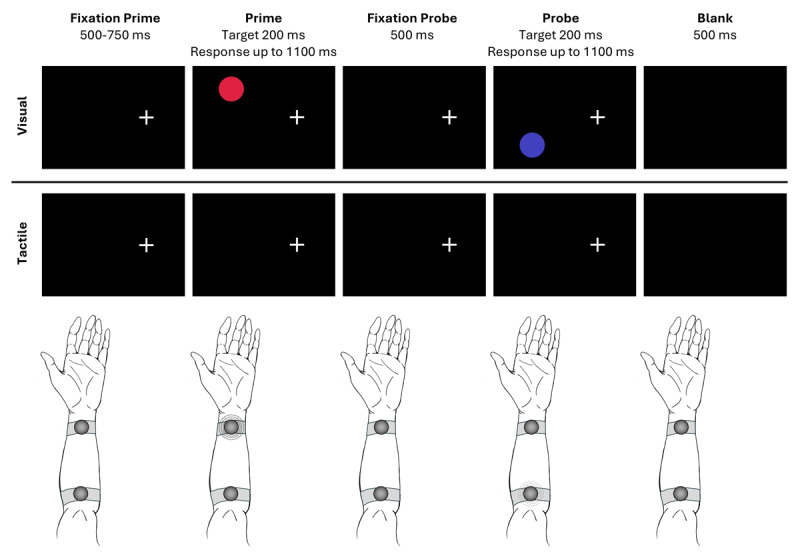
Trial sequences as used in the experiment. *Note*. Trial sequences are not drawn-to-scale. The upper row depicts the visual task, whereas the lower row depicts the tactile task. In both, the location and the non-spatial identity varies (LCFC). Here, for the visual task, the target changes from red to blue, whereas for the tactile task, the target changes from a constant vibration with high intensity to a rhythmic vibration with low intensity.

The experiment was compiled of prime-probe sequences, consisting of a prime display and a response given to it, followed by a probe display and a response given to it (see Figure 2). In both tasks, a trial started with the white fixation cross being presented vertically centered at the right screen half for 500–750 ms, which participants were instructed to fixate throughout the trial sequence.^1^ In the prime display the fixation cross was accompanied by the prime target for 200 ms. In the visual task, a red or blue target appeared on the upper left or lower left half of the screen. In the tactile task, one of the two types of vibrations appeared near the hand or the elbow of the left arm. Participants were instructed to press the spacebar with their right index finger as fast as possible. Responding was possible with target onset and up to 900 ms after target offset. Afterwards, the fixation cross was presented again in isolation at screen center for 500 ms. This was followed by the probe display which was as described for the prime display. After the probe display, a blank screen of 500 ms ended one prime-probe sequence. Missing a response during prime or probe produced an error feedback after the respective display for 1000 ms. In 20% of trials no target appeared either during the prime or probe display; here, participants were instructed to give no response and the sequence continued after 1100 ms.

In a prime-probe sequence, the location of the target could repeat (location repetition, LR) or change (location change, LC). The non-spatial feature, that is, color (red vs. blue; visual task) or type of vibration (constant, high intensity vs. rhythmic, low intensity, tactile task) could repeat (feature repetition, FR) or change (feature change, FC). These four conditions were orthogonally varied, yielding four combinations (LRFR, LRFC, LCFR, LCFC) for each modality. Combinations of location and non-spatial feature were pseudo-randomly balanced and conditions were drawn randomly. In 50% of catch trials there was no prime target but a probe target, and in the other 50% of catch trials there was a prime target but no probe target. For each modality, there were 16 practice trials drawn randomly from the pool of combinations (including catch-trials), for which participants received feedback after every response. This was followed by 256 experimental trials and 64 catch-trials. During the experimental phase, participants only received feedback for incorrect responses. Participants could take self-paced breaks after every 80^th^ trial.

### Results

#### Reaction times

All catch trials were removed from analysis. Reaction times below 50 ms or above 1.5 interquartile range above the third quartile of a participants distribution (Tukey, 1977) were excluded from analysis; for calculating the upper criterium, missed responses were excluded. Responses were only analyzed if both prime and probe responses were correct. Due to these criteria 8.36% of trials were discarded.

A 2 (modality: visual vs. tactile) × 2 (location relation: repetition vs. change) × 2 (feature relation: repetition vs. change) repeated measures ANOVA was performed on probe reaction times. There was no main effect of modality, *F*(1, 27) = 0.02, *p* = .880, η*_p_*^2^ < .01. There was a main effect of location relation, *F*(1, 27) = 9.38, *p* = .005, η*_p_*^2^ = .26, in that participants were slower for location repetitions (329 ms) compared to changes (323 ms), suggesting the occurrence of IOR. There was also a main effect of feature relation, *F*(1, 27) = 11.58, *p* = .002, η*_p_*^2^ = .30, with a benefit of feature repetition (323 ms) over change (329 ms). This effect was further modulated by modality, *F*(1, 27) = 5.71, *p* = .024, η*_p_*^2^ = .18. The interactions between modality and location, *F*(1, 27) = 3.05, *p* = .092, η*_p_*^2^ = .10, and location and feature, *F*(1, 27) = 3.06, *p* = .092, η*_p_*^2^ = .10, did not reach significance. Interestingly, the threeway interaction between modality, location, and feature became significant, *F*(1, 27) = 4.39, *p* = .046, η*_p_*^2^ = .14.

**Figure 3:**
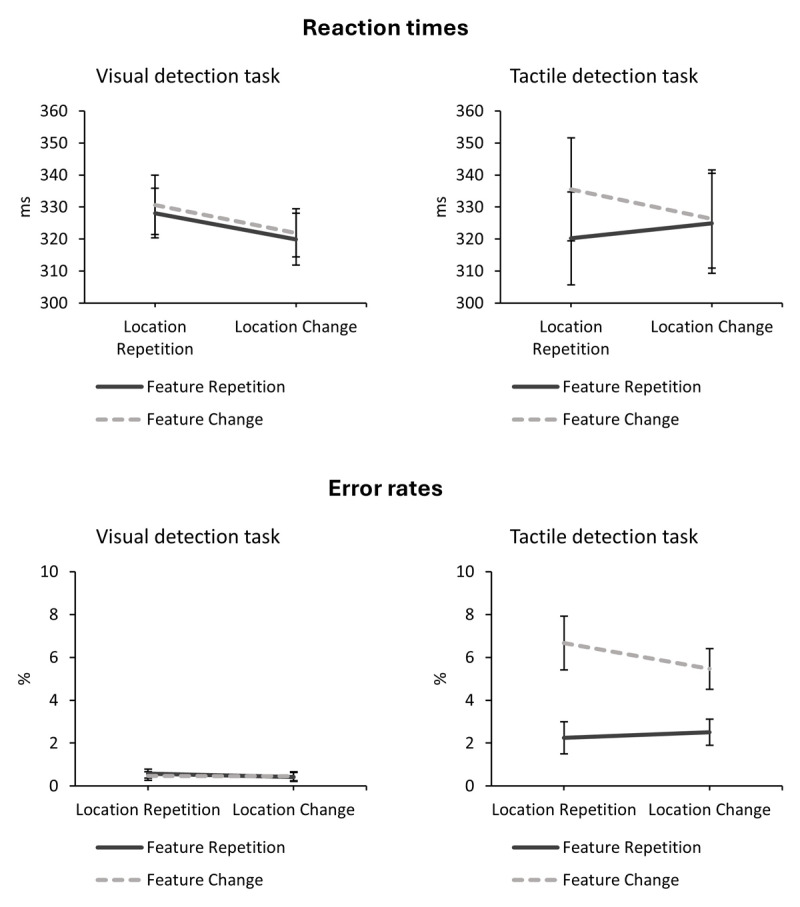
Interaction of feature relation and location relation in reaction times and error rates of the detection tasks. *Note*. Error bars represent standard errors of the mean.

Due to the significant modulations of modality on the main effect of feature as well as the interaction of feature and location, we ran separate ANOVAs for each modality. In the visual task, there was only the main effect of location relation, *F*(1, 27) = 10.12, *p* = .004, η*_p_*^2^ = .27 (LR: 329; LC: 321 ms). The main effect of feature relation, *F*(1, 27) = 2.52, *p* = .124, η*_p_*^2^ = .09, and the interaction of feature and location relation, *F*(1, 27) = 0.01, *p* = .907, η*_p_*^2^ < .01, were not significant. In the tactile task, there was a main effect of feature relation, *F*(1, 27) = 11.54, *p* = .002, η*_p_*^2^ = .30 (FR: 323; FC: 331 ms). The main effect of location relation was not significant, *F*(1, 27) = 0.97, *p* = .333, η*_p_*^2^ = .04. Interestingly, the interaction of feature and location relation became significant, *F*(1, 27) = 6.15, *p* = .020, η*_p_*^2^ = .19: Participants were slower when the feature changed (336 ms) compared to repeated (320 ms) when the location repeated; when the location changed feature repetitions (325 ms) and changes (326 ms) were comparable. The data patterns are depicted in Figure 3 (upper panel).

#### Error rates

All catch trials were removed from analysis. Error rates are the percentage of missed probe responses after correct prime responses. Thus, all trials with incorrect prime responses were excluded (2.11%).

The 2 (modality: visual vs. tactile) × 2 (location relation: repetition vs. change) × 2 (feature relation: repetition vs. change) repeated measures ANOVA on probe error rates revealed a main effect of modality, *F*(1, 27) = 36.57, *p* < .001, η*_p_*^2^ = .58, in that participants made more errors in the tactile (4.22%) compared to visual (0.48%) task. There was also a main effect of feature relation, *F*(1, 27) = 18.47, *p* < .001, η*_p_*^2^ = .41, indicating a feature repetition benefit (FR: 1.44%; FC: 3.26%). Crucially, this was modulated by modality, *F*(1, 27) = 20.72, *p* < .001, η*_p_*^2^ = .43, in that this effect was observed for tactile stimuli (FR: 2.37%; FC: 6.07%) but not for visual stimuli (FR: 0.50%; FC: 0.46%). None of the other effects became significant (all *F* ≤ 1.88; all *p* ≥ .181). The data patterns are depicted in Figure 3 (lower panel).

#### Discussion

Participants signaled the detection of visual targets repeating or changing their color and tactile targets repeating or changing their rhythm and intensity. In the tactile task, there was an overall feature repetition benefit, which is in line with the feature being bound to the response and being retrieved upon repetition (as in Schöpper & Frings, 2023). Interestingly, this further manifested in an interaction of feature relation and location relation, suggesting that binding of features irrespective of the response occurred (e.g., Hommel, 1998). All of this was absent in the visual domain.

Of note, an overall benefit for non-spatial feature repetition in the tactile task could also occur irrespective of repeating or changing the response (see Moeller & Frings, 2011), for example, caused by some type of repetition priming (for the visual domain, see, e.g., Henson et al., 2014; Kristjánsson & Campana, 2010; for the auditory domain, see, e.g., Bergerbest et al., 2004). This pattern was even pronounced for location repetitions, giving the possibility of response repetition heuristics (e.g., Pashler & Baylis, 1991) explaining the data: If information repeats, repeat the response (see also “bypass rule”, Fletcher & Rabbitt, 1978; Krueger & Shapiro, 1981). In Experiment 2 we decided to replicate the pattern of modality differences observed in Experiment 1 using a localization task. This task type has the advantage of having responses to repeat or change (Schöpper & Frings, 2024; Schöpper et al., subm.), compared to continuous response repetitions in detection tasks as used in Experiment 1. By that, we can distinguish between repetition effects irrespective of repeating or changing a response and repetition effects that interact with the response – presumed patterns of binding and retrieval.

## Experiment 2: Localization task

### Methods

#### Participants

In Experiment 1 we found a difference of binding effects between the tactile and visual domain indicated by the modulation of the main effect of non-spatial feature repetition that came with *d* = 0.45. This is similar to the difference found between the auditory and the visual domain (*d* = 0.37 in Schöpper & Frings, 2023). As S-R binding effects in auditory localization performance can be very strong (e.g., averaged *d* = 1.44 in Dyson, 2010; *d* = 1.47 in Schöpper et al., subm.), we also assumed a strong effect in the tactile localization task of at least *d* = 0.8. Thirty-two students from Trier University participated for course credit or a monetary reward of 10€ and gave written informed consent. One participant was excluded due to not following task instructions (i.e., said participant never pressed any keys, resulting in 100% missed responses). The remaining sample (26 females, four males, one other, *M*_age_ = 25.61, *SD*_age_ = 5.38; age range: 19–44) reported normal or corrected-to-normal vision. This sample sizes gives us a power of 1–ß = 1.00 for observing an effect size of at least *d* = 0.8 (α = .05, one-tailed; G*Power, Version 3.1.9.4; Faul et al., 2007).

#### Apparatus, Materials, Design, and Procedure

The experiment was identical to Experiment 1, except for the response mapping. Instead of pressing the spacebar for every target, participants were instructed to press the arrow-up key with their right middle finger in response to targets in the upper screen half (visual task) and near the hand (tactile task), and the arrow-down key with their right index finger in response to targets in the lower screen half (visual task) and near the elbow (tactile task). By that, errors were not only produced by missing a response but also by pressing the incorrect key. In such a localization task, the binding effect is derived from the interaction of feature relation and location relation.

### Results

#### Reaction times

The same exclusion criteria as reported for Experiment 1 resulted in 18.75% of trials being discarded.^2^

A 2 (modality: visual vs. tactile) × 2 (location relation: repetition vs. change) × 2 (feature relation: repetition vs. change) repeated measures ANOVA was performed on probe reaction times. There was a main effect of modality, *F*(1, 30) = 50.53, *p* < .001, η*_p_*^2^ = .63, with participants being faster in the visual (416 ms) compared to tactile (479 ms) task. The main effect of location relation did not reach significance, *F*(1, 30) = 3.56, *p* = .069, η*_p_*^2^ = .10. However, it was modulated by modality, *F*(1, 30) = 5.60, *p* = .025, η*_p_*^2^ = .16. There was a main effect of feature relation, *F*(1, 30) = 84.04, *p* < .001, η*_p_*^2^ = .74 (FR: 440 ms; FC: 455 ms), which was further modulated by modality, *F*(1, 30) = 114.65, *p* < .001, η*_p_*^2^ = .79. Feature relation and location relation interacted, *F*(1, 30) = 62.83, *p* < .001, η*_p_*^2^ = .68, depicting partial repetition costs (LRFR: 436 ms; LRFC: 467 ms; LCFR: 443 ms; LCFC: 443 ms). Importantly, this was modulated by modality, as shown by the significant three-way interaction, *F*(1, 30) = 73.44, *p* < .001, η*_p_*^2^ = .71. Due to modality modulating the main effects of feature and location relation as well as the interaction of feature and location relation, we ran separate ANOVAs for each modality.

**Figure 4:**
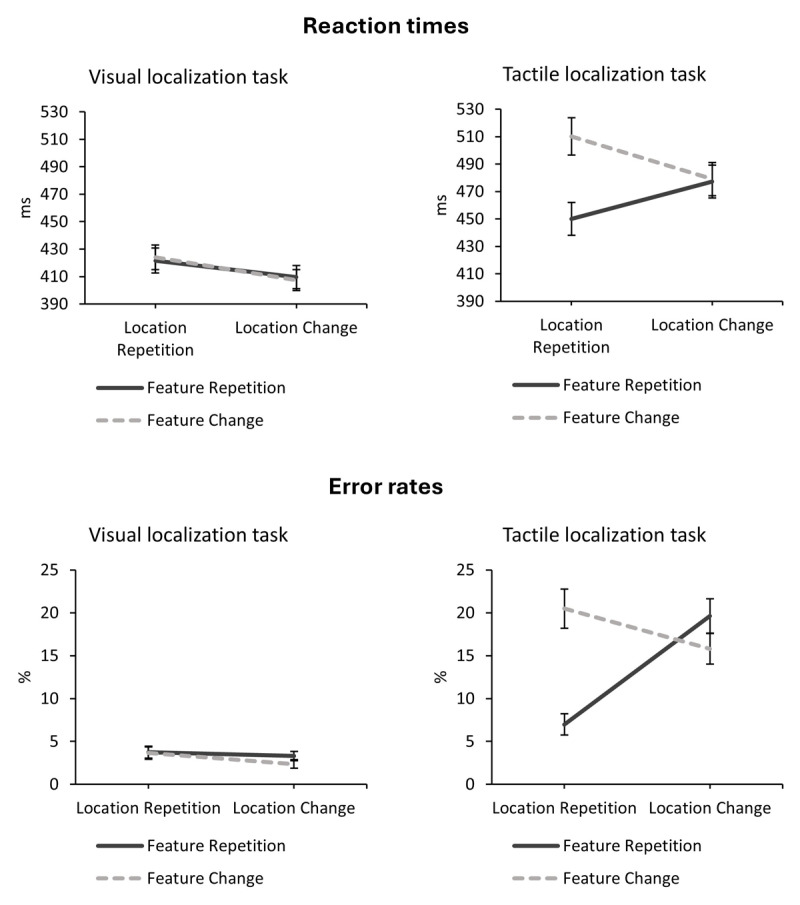
Interaction of feature relation and location relation in reaction times and error rates of the localization tasks. *Note*. Error bars represent standard errors of the mean.

In the visual task, there was only the main effect of location relation, *F*(1, 30) = 8.55, *p* = .006, η*_p_*^2^ = .23, indicating IOR (LR: 423 ms; LC: 409 ms). The main effect of feature relation, *F*(1, 30) < 0.01, *p* = .955, η*_p_*^2^ = .00, and the interaction of feature and location relation, *F*(1, 30) = 2.57, *p* = .120, η*_p_*^2^ = .08, were not significant. In the tactile task, there was a main effect of feature relation, *F*(1, 30) = 137.39, *p* < .001, η*_p_*^2^ = .82 (FR: 464 ms; FC: 495 ms). The main effect of location relation was not significant, *F*(1, 30) = 0.14, *p* = .710, η*_p_*^2^ = .01. Crucially, the interaction of feature and location relation became significant, *F*(1, 30) = 78.99, *p* < .001, η*_p_*^2^ = .73: Participants were slower when the feature changed (510 ms) compared to repeated (450 ms) when the location repeated; when the location changed feature repetitions (477 ms) and changes (479 ms) were comparable. The data patterns are depicted in Figure 4 (upper panel).

#### Error rates

Error rates are the percentage of errors made after correct prime responses. This could be due to missing a response or due to pressing the wrong key. Thus, all trials with incorrect prime responses were excluded (7.55%).

The 2 (modality: visual vs. tactile) × 2 (location relation: repetition vs. change) × 2 (feature relation: repetition vs. change) repeated measures ANOVA on probe error rates revealed a main effect of modality, *F*(1, 30) = 60.48, *p* < .001, η*_p_*^2^ = .67, in that participants made more errors in the tactile (15.72%) compared to visual (3.26%) task. There was a main effect of feature relation, *F*(1, 30) = 21.31, *p* < .001, η*_p_*^2^ = .42, with a benefit of feature repetition (8.41%) over feature change (10.57%), but the main effect of location relation did not reach significance, *F*(1, 30) = 2.90, *p* = .099, η*_p_*^2^ = .09. There was an interaction of location relation and feature relation, *F*(1, 30) = 76.48, *p* < .001, η*_p_*^2^ = .72, in line with an overall binding pattern (LRFR: 5.36%; LRFC: 12.06%; LCFR: 11.45%; LCFC: 9.08%). Modality modulated feature relation, *F*(1, 30) = 26.37, *p* < .001, η*_p_*^2^ = .47, location relation, *F*(1, 30) = 8.38, *p* = .007, η*_p_*^2^ = .22, and the interaction^3^ of feature relation and location relation, *F*(1, 30) = 54.63, *p* < .001, η*_p_*^2^ = .65.

In the visual task, there was no main effect of feature relation, *F*(1, 30) = 1.71, *p* = .201, η*_p_*^2^ = .05, or location relation, *F*(1, 30) = 1.37, *p* = .252, η*_p_*^2^ = .04. The interaction of feature and location relation was not significant, *F*(1, 30) = 1.57, *p* = .220, η*_p_*^2^ = .05. In the tactile task, there was a main effect of feature relation, *F*(1, 30) = 28.38, *p* < .001, η*_p_*^2^ = .49 (FR: 13.29%; FC: 18.15%), and a main effect of location relation, *F*(1, 30) = 6.27, *p* = .018, η*_p_*^2^ = .17 (LR: 13.74%; LC: 17.71%). Crucially, the interaction of feature and location relation became significant, *F*(1, 30) = 71.50, *p* < .001, η*_p_*^2^ = .70: When the location repeated participants made more errors when the feature changed (20.49%) compared to repeated (6.98%). When the location changed, participants made more errors when the feature repeated (19.61%) compared to changed (15.81%). The data patterns are depicted in Figure 4 (lower panel).

#### Discussion

Participants localized visual targets repeating or changing their color and tactile targets repeating or changing their rhythm and intensity. Next to an overall feature repetition benefit (as in Experiment 1), responding was heavily influenced by partial repetition costs in the tactile task, depicting a S-R binding effect. In the visual task, a binding pattern was absent.

## General Discussion

In the current study we asked participants to signal the detection (Experiment 1) and location (Experiment 2) of sequentially presented tactile stimuli. Congruent with binding approaches in action control (Frings et al., 2020, 2024; Hommel, 1998; Hommel et al., 2001) S-R binding effects emerged both in tactile detection and localization. This pattern dramatically differed from that in the visual domain. In the latter, we replicated that binding and retrieval of non-spatial features usually does not take place (e.g., Huffman et al., 2018; Schöpper & Frings, 2022, 2024; Schöpper et al., 2020, 2023). This is in line with the modality dependence for binding approaches in action control as proposed by Schöpper and Frings (2023; see also Schöpper et al., subm.). Tactile processing in detection and localization performance seems to be similar to that of auditory processing in terms of binding and retrieval (Dyson, 2010; Mondor & Leboe, 2008; Schöpper & Frings, 2023; Schöpper et al., subm.) in that binding of features defined in these domains are always bound as in discrimination tasks (see also Zmigrod et al., 2009).

Interestingly, in Experiment 1 an interaction of non-spatial feature and location emerged in the tactile condition. This suggests that both features were bound together in binary fashion (e.g., Hommel & Colzato, 2004) without any response information; such feature-feature bindings have been observed previously (e.g., Hommel, 1998). Alternatively, this pattern reflects an interplay of response repetition heuristics and inhibitory effects (see Klein, 2004; Taylor & Ivanoff, 2005; see also Christie & Klein, 2001): If the non-spatial information repeats, response repetition heuristics (e.g., Pashler & Baylis, 1991) apply in that repeating the response is fueled by repetition of intensity/rhythm. However, if the non-spatial information changes, this heuristic does not apply and IOR takes over. Lastly, it is possible that binding, retrieval, and repetition heuristics were all at play (Weissman et al., 2022).

While the absence of binding effects in visual detection and localization tasks has been attributed to the response being mapped spatially congruent with the response (e.g., Kornblum et al., 1990) thus making any resource-intensive translation from feature space to response space unnecessary (see Geissler et al., 2024; Schöpper et al., 2022a), this explanation seems to not hold for auditory or tactile targets. Detecting either of these demands the same “non-translation” as all targets irrespective of modality require the exact same response; in the same vein, localizing these is also based on direct spatial congruence – upper or lower target dot, upper or lower vibration, and left or right sound. Thus, processing auditory and tactile stimuli seems to circumvent the post-selective processing pathway (Geissler et al., 2024; Schöpper et al., 2020, 2022a; see also Hilchey et al., 2020) proposed to be necessary for observing binding effects in visual detection and localization. Given the increased error rates compared to the visual domain, it is also possible that performing detection and localization responses in the auditory and tactile tasks was overall harder, and that this increased task difficulty might lead to binding and retrieval affecting responding (see Geissler et al., 2024).

The question then emerges why this is the case. It has been found that information of the visual domain dominates information of other modalities (Colavita, 1974; Posner et al., 1976; Spence et al., 2001; for a meta-analysis, see Hirst et al., 2018). Posner et al. (1976) argued that this is the case because visual stimuli are not as alerting by themselves as stimuli from other modalities; by that, visual stimuli are only alerting if they effortfully receive attention. They argue that “if visual signals tend to evoke eye movement automatically, it may be unnecessary for them to also summon attentional systems unless the input is further classified as dangerous or interesting” (p. 169). This is completely in line with Huffman et al. (2018) who argued that retrieval-based effects are absent in visual detection and localization performance because attentional resources to non-spatial target identity are not required to give the response (see also Hilchey et al., 2018; Huffman et al., 2020; Schöpper et al., 2020). In the same vein, Schöpper and Frings (2023) and Schöpper et al. (subm.) argued that auditory stimuli are by themselves so alerting (van der Lubbe & Postma, 2005) and hard to ignore (Spence, Ranson, & Driver, 2000) that binding effects do emerge. Thus “vision’s deficient alerting capability” (Posner et al., 1976, p. 169) might be in fact the reason of absent binding and retrieval effects in visual detection and localization performance – and the presence in their auditory and tactile counterparts. To investigate this, future studies could increase the alerting capabilities of visual stimuli, for example, by associating specific target identities with high reward values (e.g., Eder et al., 2020; Theeuwes & Belopolsky, 2012) or by using stimulus categories that have been found to grab attention (e.g., arousing images, Vogt et al., 2008; faces, Theeuwes & Van der Stigchel, 2006; spiders, Mogg & Bradley, 2006; see, however, Schöpper & Frings, 2023, that face identity might not be strong enough to lead to effects of binding and retrieval).

Binding approaches in action control (e.g., Frings et al., 2020, 2024; Hommel et al., 2001) assume binding and retrieval to reflect ubiquitous processes when responding to stimuli. While their occurrence can be modulated, for example, by attention (e.g., Moeller & Frings, 2014), task instructions (e.g., Hommel et al., 2014; Memelink & Hommel, 2013), task type (Chao et al., 2022; Huffman et al., 2018; Schöpper & Frings, 2024), and target modality (Schöpper & Frings, 2023; Schöpper et al., subm.; current study), the present study suggests something potentially more far reaching: As many of the previous findings regarding the observations and modulations of binding and retrieval (for an overview, see Frings et al., 2020) have been observed with unimodal visual stimuli, these findings might be limited to the visual domain. For example, responses and features have been found to typically form binary associations (Hommel, 1998; Hommel & Colzato, 2004) while higher order interactions are very rare (but possible, see Hilchey et al., 2018; Moeller et al., 2016). It is possible that this is due to visual features being less alerting by themselves (Posner et al., 1976); thus, using auditory or tactile stimuli each defined in more than one feature dimension (e.g., intensity, frequency contrast, and temporal contrast in the auditory domain, Kayser et al., 2005) might increase the chances of observing bindings that exceed a binary structure. Further, any absent modulation of binding effects by visual information might hinge on relying on the visual domain. While at this point this remains speculation, future studies should pinpoint if modulations of binding and retrieval affect binding effects resulting from stimuli of different modalities (e.g., Zmigrod et al., 2009) to the same degree.

**Figure A1:**
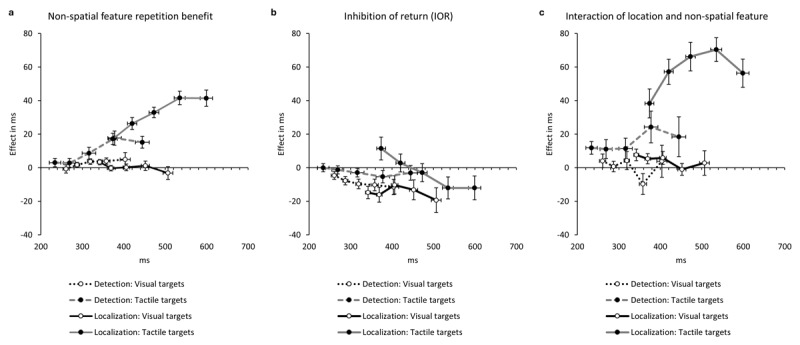
Calculated effects of non-spatial feature repetition, location repetition costs (IOR), and the interaction of location relation and non-spatial feature relation separate for each percentile and each task. *Note*. The differential values were calculated as reported in the main text. The respective differential value in ms is depicted on the y-axis and the reaction time in ms is depicted on the x-axis as a function of percentile (see also, e.g., De Jong et al., 1994; Ridderinkhof, 2002) and task. The white (visual targets) and black (tactile targets) dots represent the 10^th^, 25^th^, 50^th^, 75^th^, and 90^th^ percentile for each function separate for each task (dotted black line: visual detection, dashed grey line: tactile detection, solid black line: visual localization, solid grey line: tactile localization). Error bars represent standard error of each mean of each averaged percentile for the effect of interest (y-axis) and overall response speed (x-axis).

It has been argued that visual detection and localization performance is unaffected by retrieval as the response is executed too fast (Schöpper et al., 2020). While late responses can be affected by retrieval in different experimental designs (e.g., Chao & Hsiao, 2021; Chao et al., 2022; Schöpper & Frings, 2022, 2024; Schöpper et al., 2022b), late responding in “simple” detection and localization procedures (i.e., one stimulus on-screen demanding a response) is typically unaffected (Schöpper & Frings, 2022, 2023, 2024; Schöpper et al., 2022a; see also **Appendix A1** for the analysis of cumulative reaction time distributions of the current study). However, in the current study responding to tactile and visual stimuli was roughly the same speed in Experiment 1 – still the data pattern differed between visual and tactile targets with that in the tactile domain being in line with binding and retrieval. Further, while responding in Experiment 2 was overall faster in the visual compared to the tactile domain, even slow responses were unaffected by retrieval of non-spatial information (see **Appendix A1** and Figure A1). We thus think that response speed is not an explanation for the absence of binding and retrieval in visual detection and localization.

As a limitation, one might criticize that the rhythm of tactile targets was distinct only after 40 ms, because one stimulus identity was constant whereas the other used rhythmic alterations of 40 ms vibration and 40 ms silence. This would give a head-start for response-irrelevant information in the visual domain (see also Frings & Moeller, 2012), as colors were distinct directly with onset. However, first, the constant rhythm was also high in intensity whereas the alternating rhythm was low in intensity, making them distinct on another stimulus property. By that, one might interpret our tactile stimuli being distinct on two dimensions – intensity and rhythm – which might have fueled an effect (see Schöpper et al., 2024). Second, even if rhythm was only identified after 40 ms it still retrieved the response; thus, even potentially delayed perception of non-spatial identity allowed to start the retrieval process. Third, although both visual and tactile were relatively simple, modalities were not fully matched in terms of, for example, perceived distances or discriminability. For example, repeating or changing locations (e.g., Singh & Frings, 2020; van Dam & Hommel, 2010; see also Hommel, 2007, for response relevance of location) and salience (Schmalbrock et al., 2023) have an impact on the strength of binding and retrieval. Thus, the modality difference that arises here might be (partially) driven by the specifics of the respective visual or tactile features. Note, however, that binding effects are not observed in visual detection and localization procedures with a number of different non-spatial features (e.g., colors, Kwak & Egeth, 1992; Schöpper & Frings, 2024; face identity, Schöpper & Frings, 2023; shapes, Fox & de Fockert, 2001; Taylor & Donelly, 2002; for an overview see also Huffman et al., 2018) even if targets are made hard to perceive (Schöpper & Frings, 2024) or if displays are made more complex (direct response conditions in Geissler et al., 2024, and Schöpper et al., 2022a; see also Hu et al., 2011, 2013), suggesting that ease of selection is not the sole reason for absent effects in the visual domain.

In the current study we found evidence for binding and retrieval in tactile detection and localization tasks compared to their visual counterparts. Yet, two questions remain unanswered. First, it remains unclear if the binding pattern in the localization task (Experiment 2) emerged due to an interaction between non-spatial feature and location, between non-spatial feature and response, or both, as location and response were fully confounded. Future studies could use localization tasks with multiple target locations for each response (e.g., Schöpper et al., 2024, in the visual domain) or employ paradigms that de-confound targets and responses (e.g., S1R1-S2R2-paradigm, Hommel, 1998). The main effect of feature repetition in the detection task (Experiment 1) might be seen as tentative evidence of the non-spatial feature being bound to the repeated detection response (although, as pointed out above, this might reflect priming effects irrespective of the response). Second, it is unclear if the absence of binding effects in the visual domain is the result of a lack of binding, a lack of retrieval, or both (for a discussion, see also Schöpper et al., 2020). In some visual detection (Hu et al., 2011, 2013) and localization tasks (Schöpper et al., 2022a) non-spatial IOR arises, that is, full repetition costs; these have been discussed (Hu et al., 2011, 2013) as emerging due to a detection cost for repeated information (Lupiáñez, 2010; see also Lupiáñez et al., 2013; Martín-Arévalo et al., 2013). This suggests that at least some information is bound but, if repeated, is not “used” for retrieval.

Lastly, while non-spatial feature binding effects in the visual domain are typically absent when using detection and localization procedures, they are typically present in discrimination performance (e.g., Chao et al., 2022; Schöpper & Frings, 2024). Thus, one might muse if using a discrimination task with tactile features (and potentially also auditory features, Dyson, 2010; Schöpper et al., subm.) might boost the binding and retrieval of non-spatial features and responses/locations, for example, by non-spatial identity becoming more relevant for responding (e.g. Memelink & Hommel, 2013; see also Chao et al., 2022; Schöpper & Frings, 2024).

## Conclusion

While effects of binding and retrieval (Frings et al., 2020, 2024; Hommel et al., 2001) are typically absent in visual detection and localization performance (Huffman et al., 2018; Schöpper & Frings, 2024), a modality dependence has been proposed as S-R binding and retrieval occurs in auditory detection (Mondor & Leboe, 2008; Schöpper & Frings, 2023) and localization (Dyson, 2010; Schöpper et al., subm.). We here replicate this proposed modality dependence by showing that tactile stimuli also lead to effects congruent with S-R binding and retrieval in detection and localization performance.

## Data Accessibility Statement

Data of both experiments is available at https://doi.org/10.23668/psycharchives.21407. Code for analysis of both experiments is available at https://doi.org/10.23668/psycharchives.21408.

## Footnotes

By that, participants might have deployed some (visual) attention to the screen in the tactile task. However, we wanted to avoid that participants look around during the experiment and keep their eyes focused.

Number of exclusions was relatively high; we thus re-did the analysis on reaction times without any outlier-criteria for response speed (i.e., only criteria were prime and probe correct; 15.82% of trials were excluded based on that). The relevant interaction remained significant (*p* < .001).

Errors were made by pressing the wrong key or by missing a response. However, the three-way interaction remained significant irrespective of only analyzing wrong keypresses, *F*(1, 30) = 41.22, *p* < .001, η*_p_*^2^ = .58, or only analyzing missed responses, *F*(1, 30) = 6.25, *p* = .018, η*_p_*^2^ = .17. In the tactile task, there was a binding pattern marked by the interaction of location relation and feature relation in wrong krey presses, *F*(1, 30) = 54.34, *p* < .001, η*_p_*^2^ = .64 (LRFR: 4.77%; LRFC: 12.80%; LCFR: 14.95%; LCFC: 9.06%) and in missed responses, *F*(1, 30) = 5.13, *p* = .031, η*_p_*^2^ = .15 (LRFR: 2.22%; LRFC: 7.69%; LCFR: 4.66%; LCFC: 6.75%). In the visual task, the interaction approached but did not reach significance in wrong key presses, *F*(1, 30) = 3.95, *p* = .056, η*_p_*^2^ = .12 (LRFR: 2.59%; LRFC: 2.84%; LCFR: 3.19%; LCFC: 2.20%) but became significant in missed responses, *F*(1, 30) = 4.25, *p* = .048, η*_p_*^2^ = .12 (LRFR: 1.15%; LRFC: 0.80%; LCFR: 0.11%; LCFC: 0.16%). Although for wrong key presses the descriptive pattern of the interaction in the visual task was in line with a binding pattern and approached significance, we deem this interaction as spurious and not as an underpowered effect due to previous research showing no such pattern (e.g., Hilchey et al., 2018; Huffman et al., 2018; Schöpper & Frings, 2022, 2024; Schöpper et al., 2022a, 2024). For missed responses, the interaction is in line with a detection cost explanation (Lupiáñez, 2010; Lupiáñez et al., 2013; Martín-Arévalo et al., 2013) for non-spatial IOR (Hu et al., 2011, 2013): Target detection is impeded (in our case more often missed) if the target is identical to its previous stimulus (in identity and location); however, this IOR is reduced if the non-spatial feature changes (see also Chao et al., 2020; Hu et al., 2011), because this change eases detection. We would like to thank an anonymous reviewer for suggesting this analysis.

## Appendix A1

We analyzed cumulative reaction time distributions (e.g., Schöpper & Frings, 2024; Taylor & Ivanoff, 2005) to look at if retrieval and/or IOR emerges for later responses (e.g., Schöpper & Frings, 2022; see also Chao & Hsiao, 2021; Chao et al., 2022). After applying the cut-off criteria mentioned in the respective results section, we took the 10^th^, 25^th^, 50^th^, 75^th^, and 90^th^ percentile of probe reaction times separate for each participant for each condition in each experiment (i.e., detection task and localization task). Next, we calculated differential values for each effect of interest (as in Schöpper & Frings, 2024). The feature repetition benefit was calculated as ((LRFC+LCFC)/2)-((LRFR+LCFR)/2), with a positive value indicating a benefit for feature repetition (see Figure A1, left panel). IOR was calculated as ((LCFR+LCFC)/2)-((LRFR+LRFC)/2), with a negative value indicating a cost for location repetition, that is, IOR (see Figure A1, middle panel). The interaction of location and non-spatial feature was calculated as (LRFC-LRFR)-(LCFC-LCFR), with a positive value indicating the summed-up partial repetition costs (e.g., Schöpper & Frings, 2022, 2024) (see Figure A1, right panel). Then we conducted repeated-measures ANOVAs with Greenhouse-Geisser corrections (due to violations of sphericity) with percentile (10^th^ vs. 25^th^ vs. 50^th^ vs. 75^th^ vs. 90^th^) as the only factor on the feature repetition benefit, IOR, and the interaction term separate for each task.

### Feature repetition benefit

In the tactile detection task the effect of percentile was significant, *F*(2.815, 76.001) = 6.84, *p* < .001, η*_p_*^2^ = .20, suggesting that the non-spatial feature repetition benefit increased with increasing percentile (10^th^: 3 ms; 25^th^: 3 ms; 50^th^: 9 ms; 75^th^: 18 ms; 90^th^: 15 ms), congruent with previous research (Schöpper & Frings, 2023). In the visual detection task, the effect was not significant, *F*(2.103, 56.781) = 1.10, *p* = .344, η*_p_*^2^ = .04. In the tactile localization task, the effect of percentile was significant as well, *F*(2.643, 79.284) = 12.15, *p* < .001, η*_p_*^2^ = .29, suggesting that the effect increased with increasing percentiles (10^th^: 17 ms; 25^th^: 26 ms; 50^th^: 33 ms; 75^th^: 42 ms; 90^th^: 41 ms). The effect of percentile was not significant in the visual localization task, *F*(2.195, 65.855) = 1.50, *p* = .229, η*_p_*^2^ = .05.

### Inhibition of return

Neither in the tactile detection task, *F*(2.138, 57.735) = 0.73, *p* = .493, η*_p_*^2^ = .03, nor in the visual detection task, *F*(2.018, 54.496) = 1.58, *p* = .216, η*_p_*^2^ = .06, the effect of percentile became significant. Interestingly, in the tactile localization task the effect of percentile was significant, *F*(2.323, 69.705) = 7.463, *p* < .001, η*_p_*^2^ = .20, suggesting that at early responses a location repetition benefit occurred that turned into a cost with later responses (10^th^: 11 ms; 25^th^: 3 ms; 50^th^: –3 ms; 75^th^: –12 ms; 90^th^: –12 ms). In the visual localization task, IOR did not increase with increasing percentiles, *F*(1.683, 50.476) = 1.29, *p* = .282, η*_p_*^2^ = .04.

### Interaction of location relation and non-spatial feature relation

Neither in the tactile detection task, *F*(1.821, 49.156) = 1.02, *p* = .361, η*_p_*^2^ = .04, nor in the visual detection task, *F*(1.657, 44.736) = 1.77, *p* = .186, η*_p_*^2^ = .06, the effect of percentile became significant. In the tactile localization task, the effect was significant, *F*(2.706, 81.177) = 5.31, *p* = .003, η*_p_*^2^ = .15, suggesting that the interaction – the S-R binding effect – increased with increasing percentile (10^th^: 38 ms; 25^th^: 57 ms; 50^th^: 66 ms; 75^th^: 70 ms; 90^th^: 56 ms), congruent with previous observations (e.g., Schöpper & Frings, 2022; Schöpper et al., 2022a). The effect of percentile was not significant in the visual localization task, *F*(2.064, 61.935) = 0.71, *p* = .499, η*_p_*^2^ = .02.
